# Current State and Prospectives for Proton Boron Capture Therapy

**DOI:** 10.3390/biomedicines11061727

**Published:** 2023-06-16

**Authors:** Nhan Hau Tran, Tatiana Shtam, Yaroslav Yu Marchenko, Andrey L. Konevega, Dmitry Lebedev

**Affiliations:** 1 Petersburg Nuclear Physics Institute Named by B.P. Konstantinov of National Research Centre «Kurchatov Institute», Orlova roscha 1, Gatchina 188300, Russia; 2 Institute of Biomedical Systems and Biotechnology, Peter the Great St. Petersburg Polytechnic University, Politehnicheskaya 29, St. Petersburg 195251, Russia; 3 National Research Center “Kurchatov Institute”, Akademika Kurchatova pl. 1, Moscow 123182, Russia

**Keywords:** radiotherapy, proton therapy, proton boron capture therapy, sensitization

## Abstract

The development of new methods increasing the biological effectiveness of proton therapy (PT) is of high interest in radiation oncology. The use of binary technologies, in which the damaging effect of proton radiation is further enhanced by the selective accumulation of the radiosensitizer in the target tissue, can significantly increase the effectiveness of radiation therapy. To increase the absorbed dose in a tumor target, proton boron capture therapy (PBCT) was proposed based on the reaction of proton capture on the ^11^B isotope with the formation of three α-particles. This review summarizes data on theoretical and experimental studies on the effectiveness and prospects of proton boron capture therapy.

## 1. Introduction

Today, radiation therapy is one of the common methods in cancer treatment. Patients receive radiation treatment as an independent method, together with chemotherapy, after surgery. A favorable treatment involves a high dose load to the target area, while the dose load to adjacent normal tissues should be minimized. The various types of the radiation therapy, such as fast neutron, photon, proton, and ion therapies [1,2,3] use beam positioning and collimation to maximize the energy in the location of the tumor while keeping the mean absorbed dose in the surrounding tissue at an acceptable level.

**Figure 1:**
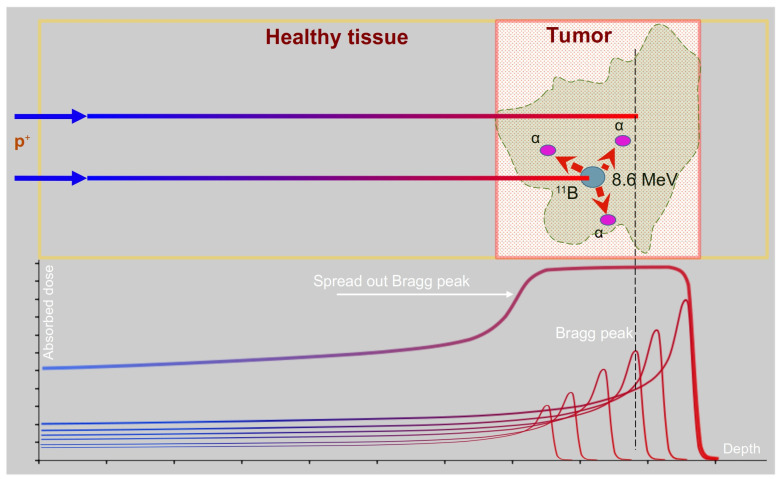
A sketch for PBCT. The dose in the tumor is enhanced by both proton beam targeting at the Bragg peak area (marked by red square) and cell targeting (increased uptake is denoted in green).

Photon radiation therapy is the most commonly used due to proven efficiency, the wide availability of high intensity sources, and the ease of precision beam positioning and dose field calculation. Proton and ion therapy, while requiring more complex and expensive equipment, have undergone rapid development in recent decades with a number of dedicated radiation facilities being built around the world and new treatment modalities being developed [4,5]. Due to the specific mechanism transferring the energy of charged particles to surrounding tissues, proton and ion therapy have a fundamental clinical advantage over conventional photon therapy. The maximum rate of energy transfer is reached at the end of the ion path, resulting in a sharply localized dose peak (Bragg peak) that rapidly falls towards zero at the distal end of the peak. The position of the Bragg peak (the depth of the location in the irradiated tissue) depends on the energy of the particles, so that it is possible to move the Bragg peak over the selected tumor volume by changing the proton energy (Figure 1).

Despite the apparent physical advantages of the proton beam over photons and the development of advanced PT modalities, such as intensity-modulated and image-guided PT [6,7], which further improve the precision of geometric targeting, a number of challenges are still to be overcome in order to exploit the full therapeutic potential of protons. [5]. Examples of these challenges include the greater sensitivity of proton dose distributions to inter- and intra-fractional variations of anatomy, as well as a large penumbra of protons. Therefore, normal tissues in close proximity to the target volume can receive a high biologically effective dose [8]. Anatomical changes in the proton pathway during a single fraction and during a course of proton therapy can significantly impair the conformity and homogeneity of the dose distribution in the target and jeopardize the preservation of normal tissues [9]. The factors mentioned above in proton therapy planning may result in a dose distribution used for treatment decisions that may differ significantly from the biologically effective dose distribution actually delivered to a patient during the course of radiation therapy.

Binary technologies that rely on an introduction into the biological environment of specific agents designed to increase the dose effects in the tumor over the normal tissue have the potential to radically increase the efficiency of radiation therapy [10]. Instead of or in addition to physical targeting, binary methods make use of the distribution of the agent that is usually designed to preferably accumulate in the tumor. The agents used in the binary methods include the radiosensitizers which accumulate specifically in the tumor cells together with radioprotectors for the normal tissue, drugs for photodynamic therapy [11] and heavy atoms for photon activation therapy [12].

One of the radiation therapy modalities relying on cell targeting is boron neutron capture therapy (BNCT). It is based on the nuclear reaction of thermal and epithermal neutrons with non-radioactive 10B nuclei that results in energetic alpha-particle production in tissue enriched with 10B isotope. According to the clinical studies, BNCT has the potential to become effective as a targeted therapy [13,14,15]. Nowadays, BNCT received a new impulse by the availability of a specialized compact neutron source specially designed for medical purposes [16]. One of the critical components for BNCT are compounds for 10B delivery to the tumor that meet the requirements for high tumor uptake, low normal tissue uptake, low toxicity and rapid clearance after treatment [17,18,19]. Currently two compounds, sodium borocaptate ((B_12_H_11_SH)Na_2_), BSH) and boronophenylalanine (C_9_H_12_BNO_4_, BPA) are commonly used for this purpose [13], allowing one to reach several-fold ratio of uptake by tumor vs. normal tissue.

The recently proposed method of proton boron capture therapy (PBCT) aims to increase the cumulative dose delivered to the target volume by the proton beam without increasing the dose load of normal tissues by employing the nuclear reaction of incident protons with boron-11 nuclei introduced into the tumor [20,21]. Using the boron-containing drugs developed for BNCT which are known to efficiently accumulate in the tumor cells, PBCT, if validated, has the potential to evolve into a treatment method that would combine the cellular biological advantages of boron neutron capture therapy and the physical targeting advantages and higher availability of proton therapy. PBCT has been primarily researched using simulation (Monte Carlo N-Particle (MCNP) and the Geometry and Tracking (GEANT4) code), but since 2018, experimental evidence has also emerged showing optimistic results in vitro. However, the theoretical prediction and experimental confirmation of radiosensitizing effects of boron are still controversial and their mechanisms remain debatable. We present a summary of theoretical and experimental data, available to date on this method.

## 2. Main Texts

### 2.1. Principles and Advantages of PBCT

First discovered by M.L.E. Oliphant and O.M. Rutherford in 1933 [22], the nuclear reaction of proton with boron nucleus has received attention as a nuclear fusion energy source due to the high amount of energy released together with a relatively high cross-section (ca. 1 mb), lack of neutron production and very small amount of gamma emission [23,24]. The main pathway of the proton boron fusion reaction (PBFR) is [20,23]:

11B + p → 12C*

12C* → 8Be + α

8Be → 2α(1)

Or, in summary

11B + p → 3 α + 8.6 MeV

PBCT’s principle of operation can be described as follows: boron is preferentially located in the tumor region that is treated using protons. When protons cross the tissue, a reaction between boron and proton will occur and generates three alpha particles. The excited carbon atom (^12^C) formed by the reaction between boron (^11^B) and proton will split into an alpha particle and a beryllium (^8^Be) atom, which subsequently divides into two alpha particles. Nearly all of the energy of this nuclear reaction is therefore released in the form of alpha-particles with energies in the range between 2 and 5 MeV [25]. These alpha particles have high linear energy transfer (LET) and a short linear path inside the tissue (under 30 µm), damaging cells in the immediate vicinity of the reaction site. This allows one to maximize the dose in the tumor region and minimize it outside by the choice of the boron compounds selectively accumulated inside the tumor. At the same time, the free path of the alpha particles is comparable to the size of a cell so that even if they are formed outside the cytoplasm due to suboptimal boron uptake, the probability of cell DNA damage remains high.

The maximum cross-section for proton boron capture reaction is reached at the proton energies around 600–700 keV (near 670 keV) and has been reported to be between 600–800 [23] and 1400 mb [25].

The fact that the reaction (1) cross section becomes significantly high at relatively low incident proton energy, i.e., around the Bragg peak region [25] implies that most of the added dose can be delivered to the tumor cells by proton beam targeting methods that are common for PT. Although a significant contribution from protons with energies up to 4 MeV (reported reaction cross-section in the hundreds of millibar) could be expected, these protons have a sub-millimeter range so that most of the alpha-particles will be released within a fraction of mm from the Bragg peak. Furthermore, in healthy tissues surrounding the tumor containing a certain amount of ^11^B compound, the formation of alpha particles would be either relatively low or completely absent, due the non-favorable local proton energy spectrum away from the tumor region. Through this combined biochemical and physical targeting, proton therapy would have a high potential to effectively target malignant cells without the surrounding tissue receiving an excess dose of radiation.

### 2.2. Simulation Studies

PBCT has first been studied using Monte Carlo N-Particle extended (MCNPX), and a series of results were published claiming a potential 50–90% increase in the deposited dose in the Bragg peak [21,26,27]. An increase in the maximum dose level by about 50% for the 80 MeV proton energy beam was reported by Yoon et al. [21]. In other simulations, the increase in the maximum dose level of PBCT has been reported between 92.4% and 96.6% [26,27]. In the early simulation study by Yoon et al. [21], a water phantom with cylindrical geometry with the diameter of 16 cm was used with a smaller cylindrical-shaped boron uptake region (BUR, purity of ^11^B—100%, density—2.08 g/cm^3^) inserted in the phantom. In the first part of the simulation, the authors examined the variation in the Bragg-peak of the proton depending on the location of the BUR for an incident 80 MeV proton beam. Second, the variation in the maximum dose level according to the proton energy was evaluated when the proton’s maximum dose level point was located in the BUR. The dose increase at the maximum point of the Bragg peak was confirmed for the proton sources of three different energies, 80, 90, and 100 MeV, with conditions identical to those in previous studies [28]. The simulation results appeared to show the basic application feasibility of PBCT. The increase in the maximum dose level reached 50.5%, 50.9%, and 79.5%, respectively, for the three energies 80 MeV, 90 MeV, and 100 MeV [21]. Moreover, the resulting maximum dose level points were almost the same as the original points. Finally, the intensity of the prompt gamma ray peak of 719 keV induced by the proton boron reaction was estimated. The simulations were performed for the water phantom with or without the BUR. The spectrum of the prompt gamma ray from the water phantom without the BUR contains no characteristic peak, while the BUR resulted in a distinct prompt gamma ray peak at 719 keV that was sufficiently high for identification [21]. This, in theory, could provide a way of monitoring the location of the Bragg peak in the boron-rich therapy site during PBCT.

This study [20] combined the GEANT4 toolkit with the mathematical method to analyze the dose distribution and estimate the dose of the PBFR method. The target volume was designated as a cube with a volume of 125 cm^3^. The normal tissue in front of the target volume was assigned a thickness of 3 cm, and the material of normal tissue was water. Fifteen pristine Bragg peaks were used to create the spread-out Bragg peak (SOBP) that was used to cover the target volume. In addition, the incident proton beams were assigned as field beams with an area of 25 cm^2^, which was identical to the face area of the target volume cube. The calculations of both the emitters of PBFR based on related cross-sections and the dose distribution of alpha emitters by using the GEANT4 toolkit showed that the absorbed dose of the target volume was enhanced due to the alpha emitters from PBFR when the ^11^B was contained in the target material. A value for the dose enhancement close to 50% was reported at the highest boron concentration used (boron molar fraction of 1.46% that corresponds to 20 wt.%). However, for a more reasonable molar fraction of ^11^B of 0.036% (5000 ppm ^11^B, which still appears very high for a therapeutically achievable concentrations), the enhancement values were less than 1.4%. The authors concluded that PBCT can be effective in enhancing the dose ratio in the target volume to the proximal region during PT [20].

Summarizing the early publications on proton boron capture in radiation therapy that used Monte Carlo simulations, a significant dose enhancement was shown in the areas where boron uptake coincided with the Bragg peak [20,21,26,27]. The mechanism responsible for the dose enhancement was suggested to be based on the production of α-particles via proton boron (^11^B) fusion reactions.

The subsequent theoretical works gave substantially lower estimates for the dose enhancement by boron-11 under PT conditions. The theoretical calculations of the PBFR rate and Monte Carlo simulations using the GEANT toolkit performed by A.Mazzone et al. [29] showed that, in the presence of 8 wt.% of ^11^B in BUR located at Bragg peak, the alpha particle production increased by less than 10^−4^ per proton and the majority of the alpha particles were produced by reactions other than p+^11^B. When scaled to the 80 ppm ^11^B content in BUR, the dose enhancement in the presence of boron was substantially less than 10^−5^ of the proton dose. The expected effect of the p+^11^B→3α reaction was therefore negligible, and the dose related to this reaction was orders of magnitude lower than the dose delivered by the primary proton beam [29].

In simulations by F. Tabbakh and N.S. Hosmane [30] using MCNPX2.7 and GEANT4, the dose enhancement effects from the ^11^B capture of protons and ^10^B capture of neutrons produced by the interaction between the proton beam and phantom were evaluated. The proton-^11^B reaction was evaluated for the entire proton spectrum. The authors reported the dose enhancement for both processes to be several orders of magnitude lower than the primary dose rate from PT when using 1000 ppm of boron.

In 2021, T.A. Chiniforoush et al. performed simulations with the MCNPX code 2.6.0 at the Snyder head phantom to evaluate the dose of proton therapy with and without boron-11 [31]. In the simulation, the tumor was defined as a cylinder with a radius of 1 cm and height of 2 cm, located at a depth of 3 cm, while the proton source was disk-shaped with a radius of 5 cm. The number of particle histories was set to 10^8^ particles, and all obtained errors were less than 10%. The boron-11 concentration outside the tumor region was set to 18 ppm, according to boron concentrations in BNCT. The effect of boron-11 accumulation in the tumor was simulated for boron-11 concentrations of 65, 500, 10^3^, 10^5^, 2.5 × 10^5^ and 5 × 10^5^ ppm. To evaluate the theoretical limit of the dose enhancement by PBFR, the whole tumor region was assumed to be filled with boron-11 (purity of boron ^11^B: 100%, density: 2.08 g/cm^3^). The authors accounted for the higher efficiency of the alfa-particles by assuming the radiation weighting factor of 20 and reporting the equivalent dose (in Sv). Modeling showed that, in the energy range from 68 to 83 MeV for an incident proton beam, where the tumor tissue for both PBCT and PT was covered with Bragg peaks, no noticeable dose increase for PBCT compared with proton therapy was observed [31]. The dose amplification was negligible at all boron-11 concentrations, and did not exceed 5% even when the entire tumor was assumed to be filled with boron-11. Finally, the yield of the prompt gamma rays at 719 keV detected in the presence of boron was reported to be less than 10^−6^ per proton.

In the recent simulation work [32] that utilized a Monte-Carlo simulation using the GEANT4 toolkit in the water phantom, the dose enhancement effect at SOBP was found not to exceed 1%, assuming that the boron uptake region consists of pure boron. For a ^11^B content in BUR of 80 ppm, the dose enhancement by the alpha particle production was estimated at 0.00008%. The summary of the simulation studies of PBCT is presented in Table 1.

### 2.3. In Vitro Studies

#### 2.3.1. Biological Effects of Boron during Proton Irradiation

The first experimental proof of PBCT biological effectiveness was published in 2018 by Cirrone et al. [34] using the human prostate cancer line DU145. There was a significant enhancement of both the cell survival and chromosome aberration effects in the presence of sodium borocaptate (BSH, Na_2_B_12_H_11_SH) when cells were irradiated with graded doses at the middle position of the 62 MeV clinical SOBP using the proton beam at the CATANA room of Istituto Nazionale di Fisica Nucleare—Laboratori Nazionali del Sud (INFN-LNS). Two different concentrations of sodium borocaptate were tested, corresponding to 40 ppm (parts per million) and 80 ppm of ^11^B. A set of DU145 cells were also grown and irradiated without BSH, as a control. The clonogenic survival fraction (SF) following irradiation with protons alone was best fitted to a linear-quadratic function of dose, i.e., SF = exp(−αD−βD^2^). Data from proton irradiation in the presence of BSH exhibited a purely exponential behavior as a function of dose. The significant effect due to boron concentration was observed: a calculated dose modifying factor (DMF) of 1.46 ± 0.12 was determined at the 10% survival level (DMF10). The BSH cytotoxicity was also studied in both DU145 cells used for clonogenic assay and MCF-10A cells used for chromosome aberrations. The enhancement of cell killing has been also studied in the presence of 80 ppm ^11^B in different positions of the SOBP. Experimental data clearly show no effect of BSH at the entrance position of the SOBP. At mid-SOBP, the fitting parameters were α = (0.31 ± 0.02) Gy^−1^ and β = (0.040 ± 0.006) Gy^−2^ for proton irradiation without BSH and α = (0.65 ± 0.02) Gy^−1^ in the presence of 80 ppm ^11^B. At the distal end of the SOBP, the BSH appeared to be more effective with a DMF of 1.75 ± 0.13 [34].

Concerning the chromosome aberrations (CA), cancer cells are known to be genetically unstable, and hence, radiation-induced chromosome rearrangements would superimpose onto an elevated confounding frequency of baseline damage, and therefore they do not lend themselves to the reliable assessment of radiation-induced DNA damage. Therefore, the non-tumorigenic breast epithelial MCF-10A cell line was chosen for scoring radiation-induced CAs [34]. When comparing BSH treated cells with cells irradiated with protons in the absence of BSH, a higher yield of all CA types was found in the former. When comparing the FISH and mFISH approach, the latter found a higher frequency because of its intrinsically greater sensitivity, as expected. Additionally, the boron-mediated enhancement of chromosomal damage is slightly amplified passing from conventional FISH scoring to mFISH analysis: the yield of CAs following 2 Gy of protons in combination with BSH increased from about 0.22 to 0.47 aberrations per cell compared to about 0.11 and 0.20. According to the assumption that protons are only slightly more effective than photons, the yield of CAs following X-rays was identical to that measured after exposure to protons in the absence of BSH. A linear–quadratic function Y = Y_0_ + D + D^2^ where the coefficient Y_0_ corresponded to the baseline CA frequency was used to fit aberration data. No statistically significant value for parameter α was found in the absence of BSH; on the other hand, in the presence of the 80 ppm of ^11^B, the values for α and β found were (0.16 ± 0.07) Gy^−1^ and (0.03 ± 0.02) Gy^−2^. DMF was calculated using the CAs number, and in a particular level set, there were 20 and 40 aberrations for 100 cells. The former resulted in a DMF of 2.1, while the latter was calculated as 1.6. These results support the notion that the presence of BSH results in a significant dose-modifying effect on proton irradiation, increasing cell lethality and DNA damage, consistent with the action of the alpha particles produced by proton–boron fusion [34].

#### 2.3.2. Dependency of Enhancement Effects of Boron on the Proton Energy

Continuing the research work, in 2021, the same group of researchers, Bláha et al., tested PBCT at a 250 MeV proton beam used for deep-seated cancers at the Centro Nazionale di Adroterapia Oncologica (CNAO), Pavia, Italy [35]. They used Sodium Mercapto Dodecaborate (BSH) as an ^11^B carrier, they evaluated cell killing on DU145 prostate cancer cells and quantified chromosomal aberrations (CA) by FISH painting and DNA repair pathway protein expression by Western blotting on non-cancer epithelial breast MCF-10A cells. In previous experiments [34], they only evaluated the enhancement of BSH-induced DNA damage as CAs on the non-tumorigenic MCF-10A cell line when irradiating samples at the SOBP mid position of the 62 MeV proton beam of INFN-LNS. Additionally, in this study [35], the authors irradiated cells at three locations, i.e., the entrance, middle and distal SOBP, corresponding to water depths of 1.23.76 and 29.45 mm, respectively. Major LET values of 1.58, 5.02 and 16.32 keV/mm are relevant. In addition, the expression of the DNA damage-activated repair proteins was also investigated in the middle of SOBP. The study results show that, at the distal position, the samples treated with BSH had a greater CA yield than the samples without the boron carrier under proton irradiation, while this was not observed at the beam entrance [24]. Furthermore, a larger CA frequency was also observed at the distal SOBP site compared with previously recorded results at the middle SOBP in BSH-treated MCF-10A cells. Moreover, this result, which demonstrates a depth-dependent increase in the BSH-mediated enhancement of clonogenic cell killing in DU145 cells, corroborates the view that high-LET a-particles from the p-B reaction are the most likely underlying mechanism for explaining the appearance of complex CAs in irradiated BSH-treated cells.

Additionally, the research results [35] also show the influence of the boron carrier BSH on the DNA repair process under the effect of a proton beam: first, the expression of the Ataxia Telangiectasia and Rad3-Related kinase (ATR), a protein activated upon double strand breaks (DSB) formation, increased at 30 min after irradiation (2 Gy) in BSH-treated samples and it decreased at 24 h after irradiation in samples without BSH, which was still higher than in samples pretreated with BSH [35]. Then, Ku70 protein, which is a marker of non-homologous recombination activation, was also increased 30 min after irradiation in the presence of BSH, suggesting that the non-homologous end joining pathway (NHEJ) is also activated in response to DSB formation, such as homologous repair [36]. Like ATR, Ku70 levels at 24 h post-irradiation remained high, with and without BSH pretreatment, for controls. It is these processes that explain the results’ CA analysis, due to the error-prone DSB machinery, especially of the NHEJ. In addition to the double-strand break repair system, there is also the single-strand break (SSB) repair system and they both work at the same time, contributing to DNA damage response. Additionally, base excision repair (BER) is one of the major pathways involved in the repair of SSB sites, among which the most important enzyme is the polymerase beta (POLB) [37]. Unexpectedly, nucleotide excision repair (NER), and its major regulator Xeroderma Pigmentosum Group A-Complementing Protein (XPA), did not show expression levels correlated with BSH treatment.

The authors also used a high-energy proton beam stream to irradiate the DU145 cell at three depths, corresponding to the beam entrance as well as the mid and distal positions, along the clinical 180 mm SOBP at the Clinical CNAO Proton Beamline [35]. The results obtained were like those for the low-energy beam [34]: the presence of BSH resulted in enhanced radiation-induced cell death at the mid- and distal SOBP positions, whereas it was not observed for the sample which is irradiated at the beam entrance. However, the magnitude of the radiation sensitization effect due to the p-B response in CNAO was slightly smaller than that in INFN-LNS which could be explained by the higher overall energy distribution of the incident proton beam along the SOBP. The authors also investigated proton-induced DNA damage in MCF-10A cells irradiated along the CNAO proton SOBP enhanced in the presence of BSH. Similar values for both the overall CA frequency and that of complex-type CAs were found for the samples irradiated at the entrance and the mid-SOBP positions, whereas lower values of all types of CAs, particularly those of complex ones, were observed at the distal SOBP position of the CNAO beamline when compared with low-energy irradiation.

#### 2.3.3. Radiosensitizing Effects of Boron Compounds during Gamma and Proton Irradiation

In the recent work by Shtam et al. [38], the radiosensitizing effect of BSH on glioma cells during proton irradiation near the Bragg peak was estimated for different set-ups using proton beams with an initial energy of 80 MeV and 200 MeV. No significant effect of boron-11 concentrations up to 160 ppm on cell survival and colony formation was reported for either of the set-ups. Furthermore, no significant effect was reported for DU145 cells (used in the work 13), when they were irradiated at either the distal or proximal end of the Bragg peak, even when the boron-11 concentration was raised to 250 ppm.

Another work by Manandhar et al. investigated the effect of ^11^B compounds on DU145 cells subjected to proton and X-ray radiation, including clonogenic survival rate and the double-stranded breaks frequency observed by γH2AX and 53BP1 foci [39]. BSH and BPA were used as ^11^B carriers in contraindications corresponding to ca.80 ppm of ^11^B. No effect of boron on the frequency of double-stranded breaks was observed in cells irradiated by protons with the mean energy of 60.5 MeV and of 7.6 MeV near the Bragg peak. At the same time, the authors reported a slight radiosensitizing effect of BSH on DU145 cells which appeared to be independent of proton energy and was also observed for X-ray irradiation. No change in the double-stranded breaks implies that alpha particle production via PBFR has a negligible effect on the biological effectiveness of proton irradiation, whereas the radiosensitizing effect of BSH is probably unrelated to DNA damage.

The described radiosensitizing effect of boron-11 could be a consequence of the biochemical properties of the boron-containing compound. For example, the radiosensitizing effect of the boron containing compound (sodium tetraborate, Na_2_B_4_O_7_) on the viability of cancer cells subjected to proton irradiation was detected in vitro in two human malignant glioma cell lines [40]. However, a similar radiosensitizing effect of sodium tetraborate was seen for A172 cells when subjected to gamma-radiation. The observed decrease in the survival rate of cells pre-incubated with sodium tetraborate was therefore likely caused by the pharmacological or chemical properties of this compound rather than dose enhancement via PBFR [40].

#### 2.3.4. Possible Mechanisms of Radiosensitizing Effects of Boron

While early simulations and some of the experimental studies appear to provide convincing evidence for the effectiveness of proton boron capture therapy, the degree of the radiosensitizing effect and its mechanism is far from being established. The effect of BSH on the cell survival appears significant in the experimental studies [34,35], but the influence of the p+ ^11^B reactions remains speculative. The computer simulations [20,21,29,30,31,32,33] indicate that, at a concentration of BSH, corresponding to 80 ppm ^11^B, the probability of a reaction was by several orders of magnitude too low to bear any effects of therapy. It can be hypothesized that this is only true at the macroscopic level, but at the cellular level, the dose ratio might strongly favor proton-induced reaction on boron. However, the use of BSH or similar boron carriers (such as boronophenylalanine, BPA) in boron neutron capture therapy can only achieve a several-fold gain of boron concentration in the tumor tissues, relative to the healthy ones. Therefore, it seems highly unlikely that the decrease in cellular survival probability and larger cellular damage reported by Cirrone et al. in [34,35] in the presence of BSH is caused by the dose enhancement by p+^11^B→3α nuclear reaction.

One of the explanations for the apparent discrepancy between simulation and the experiment that was proposed by Cirrone et al. and Bláha et al. [34,35] was the higher effectiveness of the alpha-particles produced by the nuclear reaction in killing the cancer cell via double-strand DNA damage and chromosome aberrations. Heavy ions are indeed known to have a higher relative biological effectiveness (RBE) than photons and protons, but it still seems to be orders of magnitude lower than that required to account for the results reported. Moreover, as pointed out in [29], the alpha-particle production under typical proton irradiation conditions is about 10^4^ per cm^3^, which is several orders of magnitude lower than the typical cell concentration. Considering the alpha-particle range of under 30 um, the upper estimate of the volume subjected to excess radiation would be less than 0.5% of the total sample volume. Thus, only a very small fraction of the irradiated cells could possibly be traversed by alpha-particles. It is therefore unlikely that a therapeutic effect could be reached, unless a large gain in the boron concentration in the tumor is achieved (orders of magnitude higher than that which could be used in therapy based on the toxicological limits).

Another hypothesis put forward by the same authors was that radiosensitizing effect of BSH that could be attributed to the indirect effects of the alpha irradiation, such as radiation-induced bystander effects (RIBEs) [41]. These effects were reported in several works, particularly with respect to alpha-particle irradiation. H. Nagasawa and J.B. Little [42,43] reported a high rate of mutagenesis in Chinese hamster ovary cells when exposed to a relatively low flux of alpha-particles. With the fraction of the cell nuclei transversed by alpha-particles kept as low as 5% or even 1%, the authors claimed that the observed rate could only be explained by mutations in bystander cells not exposed to direct ionization damage. Similar results on chromosomal instability were reported by S.A. Livermore et al. in bystander bone marrow cells [44] and were also seen in keratinocytes and fibroblasts [45]. While the exact mechanisms and the universal nature of RIBE are still far from clear [46], it could conceivably explain the experimental observations of the apparent damage to many cells which could not be directly affected by the sparse alpha-particle flux produced by proton–boron fusion.

## 3. Conclusions

Despite the optimistic experimental results, the mechanisms for the radiosensitizing effect of boron compounds during proton irradiation remain unclear and probably involve the biochemical effect of BSH and/or bystander effects induced by the alpha-particle irradiation of a fraction of the cells rather than dose enhancement via PBFR. These effects likely to be sensitive to the conditions of growth and treatment of the cell culture or irradiated tissue. Future investigation into the mechanisms of the enhancement of proton radiation effectiveness by the presence of boron reported in the works [34,35,39,40] is required, as it could have implications not only for PBCT prospectives but also for the further development of other treatment methods which are based on boron nuclear reactions, including BNCT and Neutron capture enhanced proton therapy, the new emerging PT modality.

## Figures and Tables

**Table 1:** The reported dose enhancement from boron compounds in PT simulations, scaled to 100 ppm ^11^B concentration.

Source	Software	Dose Enhancement per 100 ppm ^11^B
Jung et al., 2017 [26]	MCNPX	5–9%
Yoon et al., 2014 [21], Adam et al., 2016 [33], Liu et al., 2019 [20]	MCNPX, GEANT	0.01–0.03%
Mazzone et al., 2019 [29], Tabbakh and Hosmane, 2020 [30], Chiniforoush et al., 2021 [31], Meyer et al., 2022 [32]	MCNPX, GEANT	0.00005–0.0004%

## Data Availability

Not applicable.
